# MBNL3 Acts as a Target of miR-302e to Facilitate Cell Proliferation, Invasion and Angiogenesis of Gastric Adenocarcinoma via AKT/VEGFA Pathway

**DOI:** 10.4014/jmb.2401.01027

**Published:** 2024-05-30

**Authors:** Weiping Tang, Can Huang, Bing Jiang, Junjun Lin, Yecai Lu

**Affiliations:** Department of Gastrointestinal Surgery, Chaohu Hospital of Anhui Medical University, Chaohu 238000, Anhui, P.R. China

**Keywords:** MBNL3, gastric adenocarcinoma, AKT/VEGFA pathway, miR-302e

## Abstract

Gastric adenocarcinoma (GAC) is a common, malignant type of tumor in human, and is accompanied with higher mortality. Muscleblind-like 3 (MBNL3) was found to be a pivotal participator in aggravating this cancer’s progression. However, the regulatory effects of MBNL3 on GAC development have not been investigated. We therefore sought to study the functions of MBNL3 in GAC progression. In this study, it was demonstrated that MBNL3 exhibited higher expression, and GAC patients with higher MBNL3 expression had poor prognosis. Overexpression of MBNL3 facilitated, and knockdown of MBNL3 suppressed cell proliferation, invasion, and angiogenesis in GAC. Further experiments showed that miR-302e targets MBNL3. Rescue assays then uncovered that the miR-302e/MBNL3 axis aggravated GAC progression. In addition, MBNL3 activated the AKT/VEGFA pathway, and the suppressive regulatory impacts of MBNL3 knockdown on GAC cell proliferation, invasion, and angiogenesis could be rescued after 740 Y-P treatment. Through in vivo assay, it was proved that MBNL3 accelerated tumor growth in vivo. In conclusion, MBNL3 acted as a target of miR-302e to facilitate cell proliferation, invasion, and angiogenesis of gastric adenocarcinoma through the AKT/VEGFA pathway. Our findings illustrate that MBNL3 may be an available bio-target for GAC treatment.

## Introduction

Gastric carcinomas are a hackneyed type of cancer, and represent the principal cause of cancer-associated death [1, 2]. Gastric adenocarcinoma (GAC) is a subtype of gastric cancer, with an incidence accounting for about 95%of all gastric malignancies [3]. While the techniques of endoscopic technology, surgery, chemotherapy, and radiotherapy have all improved [4, 5], the current treatments for GAC are still limited and the mortality rate remains high. Therefore, discovering the molecular biomarkers and their regulatory mechanisms underlying the development of GAC is vital for accurate diagnosis, early intervention, and resultful treatment.

Muscleblind-like 3 (MBNL3) is an oncofetal splicing factor. Recent reports revealed that MBNL3 expression is dysregulated in diversified cancers, thereby affecting tumor growth and development. For instance, higher expression of MBNL3 is positively associated with PTX resistance and results in poor prognosis in ovarian cancer [6]. In addition, MBNL3 affects cell invasion in pancreatic ductal adenocarcinoma [7]. Furthermore, in non-small cell lung cancer, the lncRNA SBF2-AS1/miR-302a/MBNL3 axis modulates radiosensitivity and tumorigenesis [8]. Moreover, the MBNL3 splicing factor heightens the PXN expression to aggravate the progression of hepatocellular carcinoma [9]. Nevertheless, the functions and regulatory mechanism of MBNL3 in GAC progression remain unveiled, and therefore needed to be further investigated. AKT (protein kinase B, a serine/threonine kinase) can regulate metabolism, cell growth, angiogenesis, and other biological processes [10]. AKT is a downstream signal for phosphatidylinositol 3-kinase (PI3K) [11]. The PI3K/AKT pathway has been demonstrated to be a pivotal pathway in gastric cancer [12, 13]. However, the regulatory effects of MBNL3 on the AKT pathway in GAC have not been probed.

Here, we sought to probe the regulatory impacts of MBNL3 on the malignant behaviors of GAC. Our results revealed that MBNL3 exhibited higher expression, and MBNL3 acted as a target of miR-302e to facilitate cell proliferation, invasion, and angiogenesis of gastric adenocarcinoma through the AKT/VEGFA pathway. This work may provide a helpful bio-target for GAC treatment, and help relieve the suffering of GAC patients.

## Materials and Methods

### Sample Tissues

Three paired GAC tissues and adjacent normal tissues from Chaohu Hospital of Anhui Medical University were utilized for our work. These GAC patients have accepted no treatment, and signed the informed consents. This work was approved by the Ethics Committee of Chaohu Hospital of Anhui Medical University (KYXM-202302-006). The obtained tissues were kept in liquid nitrogen for further experiments.

### Cell Lines and Culture

The normal human gastric epithelial cell line GES-1 and GAC cell lines (AGS and MKN45) were purchased from American Tissue Culture Collection (ATCC, USA). Cell culturing was conducted using DMEM medium (Sigma-Aldrich, USA) at 37°C in a humid incubator with 5% CO_2_.

### Cell Transfection

The pcDNA3.1 plasmids targeting MBNL3 (MBNL3) with negative control (Vector) and shRNAs targeting MBNL3 (shMBNL3#1, shMBNL3#2, shMBNL3#3) with negative control (sh-NC) were generated by GenePharma (China). The transfection of these constructed plasmids into GAC cells was carried out using Lipofectamine 2000 (Invitrogen, USA).

### Western Blot

The proteins were isolated from GAC cells. The separation of proteins was conducted with 10% SDS-PAGE, after which the proteins were transferred to PVDF membranes (Beyotime, China). Post blocking, the primary antibodies were mixed into membranes for a 12 h incubation period at 4°C followed by washing. Subsequently, the appropriate secondary antibodies (1:2000; ab7090) were mixed for another 2 h followed by washing. Then, the assessment of the bands was performed using a chemiluminescence detection kit (Thermo Fisher Scientific, USA). The primary antibodies were: MBNL3 (1:1000; ab197590; Abcam, China), p-PI3K (1 μg/ml; ab3607), PI3K (1 μg/ml; ab3607), p-AKT (1 μg/ml; ab3607), AKT (1 μg/ml; ab3607), VEGFA (1 μg/ml; ab3607) and β-actin (the internal reference) (1:1000; ab39670).

### CCK-8 Assay

The Cell Counting Kit-8 (CCK-8; Dojindo Laboratories, Japan) was utilized in this assay. The 96-well plate was plated with GAC cells (1000 cells/well). At 0, 24, 48, 72 h, each well was filled with the CCK-8 solution (10 μl) for another 4 h. Lastly, the absorbance (at 450 nm) was measured under a spectrophotometer (Thermo Fisher Scientific).

### Transwell

Transwell chambers (8 μM, Corning, USA) coated with Matrigel (BD Biosciences, Franklin Lakes, USA) were used in this assay. The GAC cells and serum-free medium (200 μl) were supplemented into the upper chamber, and following that, the medium with 20% FBS (600 μl) was supplemented into the lower chamber. After 48 h, fixation (4% paraformaldehyde) and dyeing (0.1% crystal violet) were performed. Lastly, the invaded cells were counted via a microscope (Leica, Germany).

### Tube Formation Assay

A Matrigel-coated 24-well plate with transwell inserts (8 μm pore size, USA) was employed for this assay. First, HUVECs (1 × 10^5^ cells) were put into the plate, and then the cells were mixed with the conditional culture medium (100 μl) from transfected GAC cells (Vector, MBNL3, sh-NC, sh-MBNL3 and sh-MBNL3+740 Y-P) for 24 h. Eventually, the tube formation was evaluated under a microscope.

### EdU Assay

The Cell-Light EdU DNA Cell Proliferation Kit (RiboBio, China) was employed for this assay. The GAC cells were mixed with EdU (50 μM) for 2 h. After being treated with 4% paraformaldehyde and 0.5% Triton-X-100, the GAC cells were stained with Apollo dye solution and 4',6-diamidi-diamidino-2-phenylindole (DAPI). Lastly, counting of the EdU-positive cells was conducted under a fluorescence microscope (Leica, Germany).

### RT-q PCR

TRIzol reagent (Invitrogen, USA) was adopted for separating RNAs from GAC cells. Then, the SuperScript II Reverse Transcriptase Kit (Invitrogen) was utilized for transcribing RNA to cDNA. The qRT-PCR was executed using the SYBR Premix Ex Taq Kit (Takara, China). The mRNA expression was determined under the 2^−ΔΔCt^ method.

### Luciferase Reporter Assay

The binding ability between miR-302e and MBNL3 was confirmed. The wild-type sequences (CACCUUUUU U**GCACUU**A) or mutant-type sequences (CACCUUUUUU**CGUGAA**A) of MBNL3 (the binding sites for miR-302e) were put into the pmirGLO dual-luciferase vectors (Promega, USA) to construct the MBNL3-WT and MBNL3-MUT reporter vectors. The miR-302e mimic/NC mimic was co-transfected with these reporter vectors into GAC cells using Lipofectamine 2000. The luciferase activity was assessed with the dual-luciferase reporter assay system (Promega) after 48 h.

### In Vivo Assay

Male BALB/c nude mice (4-week-old, *n* = 6, Vital River Co., China) were stochastically divided into 2 groups (*n* = 3 mice/group). The transfected AGS cells (sh-NC or sh-MBNL3) at the concentration of 5 × 10^6^ cells/mouse were injected into the right flanks of mice. Post one month, the tumor growth (size, volume and weight) was evaluated. This work (VS212601457) was approved by the Animal Care and Use Committee of Beijing Viewsolid Biotechnology Co., Ltd.

### IHC Assay

The GAC tissues were immobilized in 4% PFA and embedded in paraffin. Next, a microtome (Leica) was utilized to cut specimens into 4 μm sections. After dewaxing, re-hydration, and washing, the sections and primary antibody (Ki-67) were mixed at 4°C for 12 h. After another washing, the sections were added with secondary antibody (1:1000, ab6721, Abcam, China). Then, staining of the sections with diaminobenzidine (DAB) and re-staining with hematoxylin were performed. Lastly, IHC images were captured using a microscope (Nikon, Japan).

### Statistical Analysis

The data were expressed as the mean ± SD. SPSS 22.0 statistical software (IBM Corp., USA) was utilized to perform the statistical analysis. All experiments were conducted in triplicate. The comparisons were analyzed by Student’s *t*-test or one-way analysis of variance (ANOVA) (for two or more groups). *p*<0.05 was deemed statistically significant.

## Results

### MBNL3 Owned Higher Expression in GAC

**Fig. 1:**
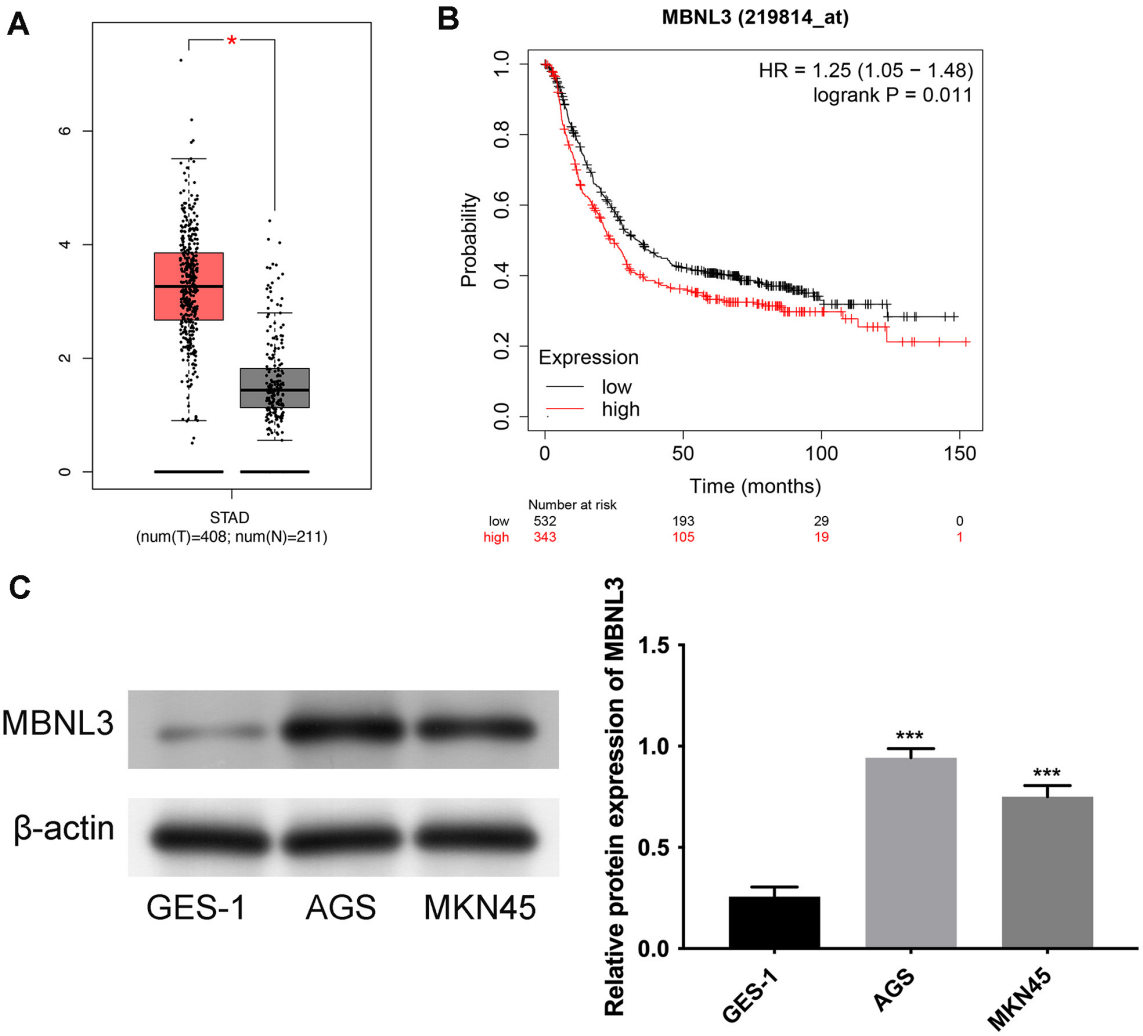
MBNL3 exhibited higher expression in GAC. (**A**) The mRNA expression of MBNL3 was confirmed in normal tissues and STAD tissues through GEPIA database. *p < 0.05. (**B**) The prognosis of GAC patients with high or low MBNL3 expression was verified through KM-plotter online database. *p* = 0.011. (**C**) The protein expression of MBNL3 was detected in normal human gastric epithelial cell line GES-1 and GAC cell lines (AGS and MKN45). ***p < 0.001.

As shown in Fig. 1A, MBNL3 mRNA levels were higher in STAD tissues than that in normal tissues. Additionally, GAC patients with higher MBNL3 expression had a worse prognosis (Fig. 1B). Moreover, MBNL3 exhibited higher expression in GAC cell lines (AGS and MKN45) than that in a normal human gastric epithelial cell line (GES-1) (Fig. 1C). These data demonstrate that MBNL3 exhibited higher expression in GAC.

### Overexpression of MBNL3 Facilitated Cell Proliferation, Invasion, and Angiogenesis in GAC

**Fig. 2:**
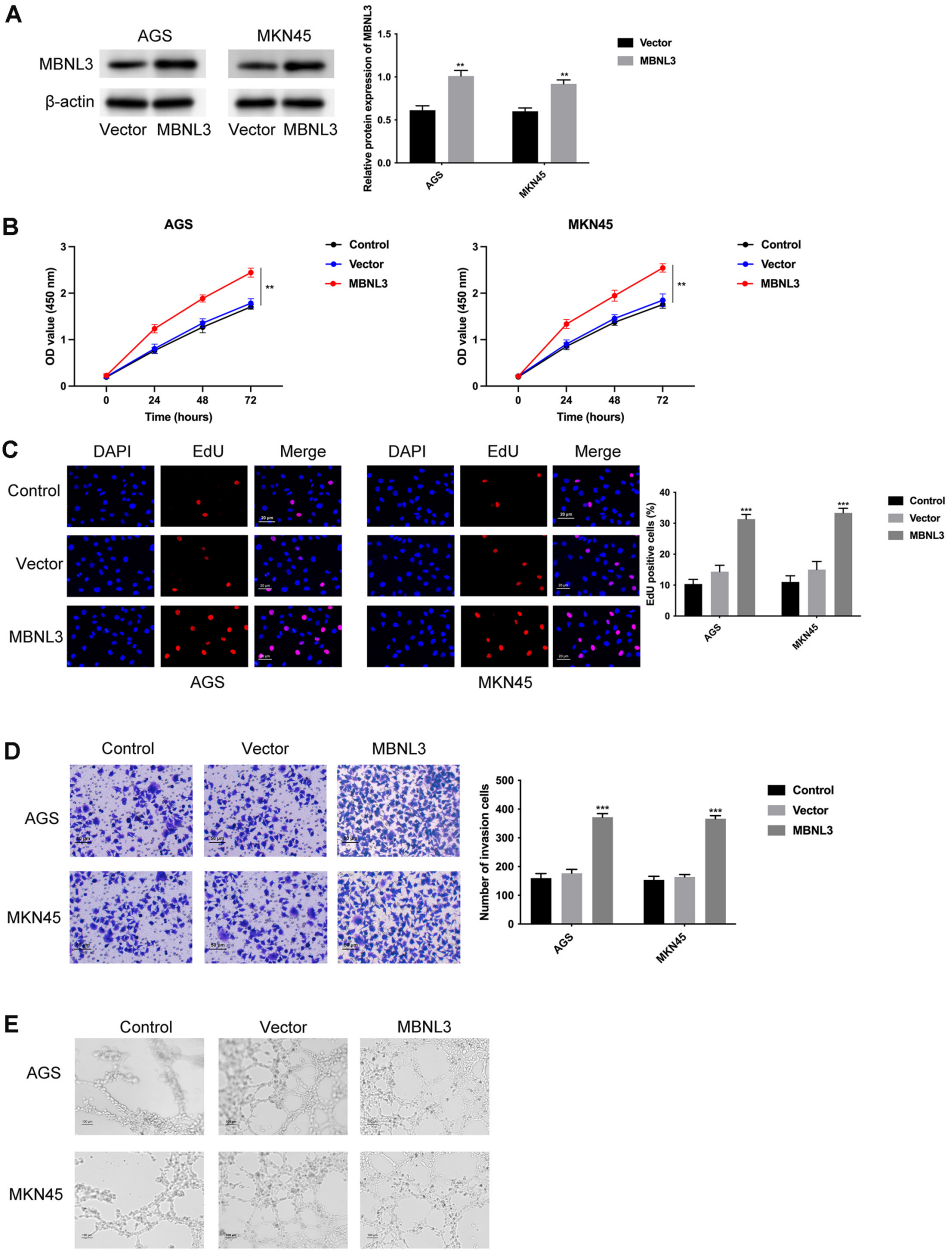
Overexpression of MBNL3 facilitated cell proliferation, invasion, and angiogenesis in GAC. (**A**) The overexpression efficiency of MBNL3 was tested in AGS and MKN45 cells through western blot. (**B**) The cell proliferation ability of AGS and MKN45 cells was determined through CCK-8 assay. (**C**) The cell proliferation was assessed through EdU assay. (**D**) The cell invasion was evaluated through Transwell assay. (**E**) The angiogenesis ability was assessed through tube formation assay. **p < 0.01, ***p < 0.001.

Firstly, the overexpression efficiency of MBNL3 was confirmed by western blot assay (Fig. 2A). The AGS and MKN45 cell proliferation were strengthened after overexpressing MBNL3 (Fig. 2B and 2C). Furthermore, the cell invasion was increased after overexpressing MBNL3 (Fig. 2D), and the angiogenic ability was also enhanced after MBNL3 overexpression (Fig. 2E). Taken together, overexpression of MBNL3 facilitated cell proliferation, invasion, and angiogenesis in GAC.

### Repression of MBNL3 Retarded Cell Proliferation, Invasion, and Angiogenesis in GAC

**Fig. 3:**
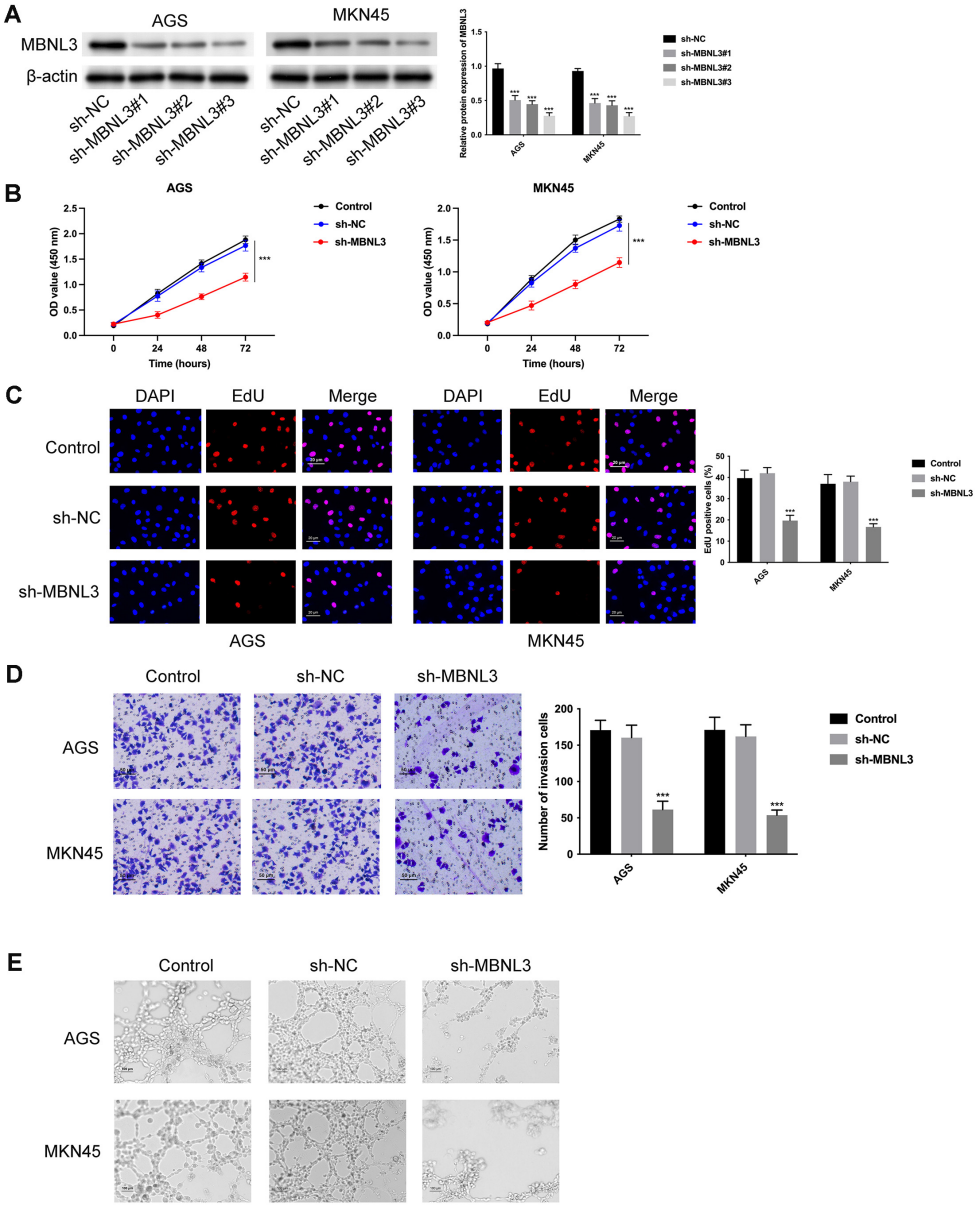
Knockdown of MBNL3 suppressed cell proliferation, invasion, and angiogenesis in GAC. (**A**) The knockdown efficiency of MBNL3 was confirmed through western blot. (**B-C**) The cell proliferation ability was detected through CCK-8 and EdU assays. (**D**) The cell invasion was tested through Transwell assay. (**E**) The angiogenic ability was examined through tube formation assay. ***p < 0.001.

The knockdown efficiency of MBNL3 was verified in Fig. 3A, while cell proliferation was reduced after silencing MBNL3 (Fig. 3B and 3C). Moreover, cell invasion was attenuated after MBNL3 knockdown (Fig. 3D). The angiogenic ability was weakened after MBNL3 suppression (Fig. 3E). These findings suggested that knockdown of MBNL3 suppressed cell proliferation, invasion, and angiogenesis in GAC.

### MiR-302e Targeted MBNL3 to Modulate GAC Progression

**Fig. 4:**
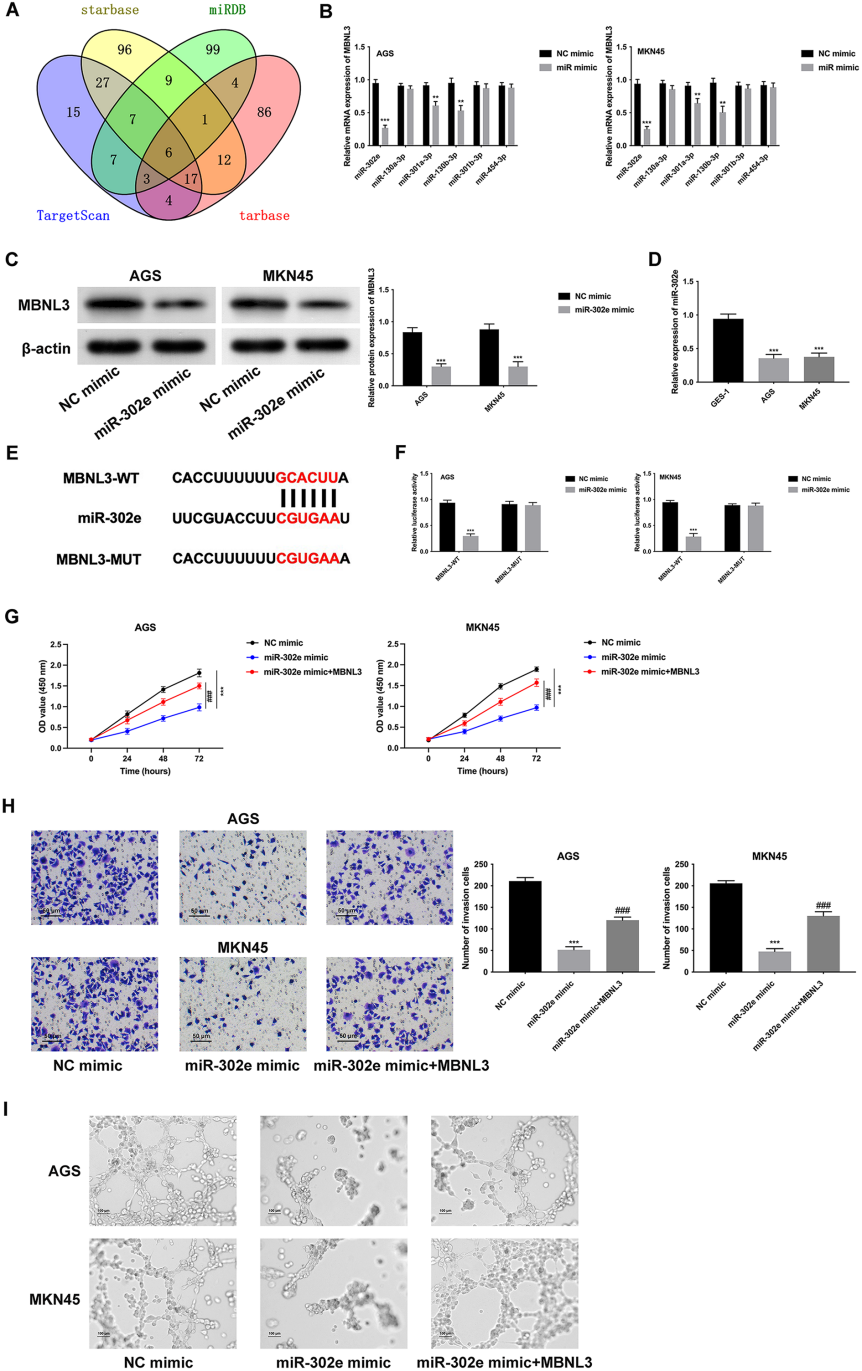
miR-302e targets MBNL3 to modulate GAC progression. (**A**) The miRNAs (miR-302e, miR-130a-3p, miR- 301a-3p, miR-130b-3, miR-301b-3p and miR-454-3p) that can combine with MBNL3 were confirmed through intersecting "TargetScan," "starbase," "miRDB," and "tarbase" online databases. (**B**) The MBNL3 mRNA expression was examined after overexpressing these miRNAs (miR-302e, miR-130a-3p, miR-301a-3p, miR-130b-3, miR-301b-3p and miR-454-3p). **p < 0.01, ***p < 0.001. (**C**) The MBNL3 protein expression was determined after overexpressing miR-302e. ***p < 0.001. (**D**) The miR-302e expression was tested through RT-q PCR. ***p < 0.001. (**E**) The binding sites (WT or MUT) of MBNL3 for miR- 302e and were shown. (**F**) The binding abilities among miR-302e and MBNL3 were assessed through luciferase reporter assay. ***p < 0.001. (**G**) The cell proliferation was evaluated through CCK-8 assay. (**H**) The cell invasion was examined through Transwell assay. (**I**) The angiogenic ability was evaluated through tube formation assay. Groups were separated into the NC mimic, miR-302e mimic and miR-302e mimic+MBNL3 group. ***p < 0.001 vs the NC mimic group; ###*p* < 0.001 vs. the miR- 302e mimic group.

There are 6 miRNAs (miR-302e, miR-130a-3p, miR-301a-3p, miR-130b-3, miR-301b-3p and miR-454-3p) that can combine with MBNL3 through intersecting TargetScan, starbase, miRDB, and tarbase online databases (Fig. 4A). In addition, the MBNL3 mRNA expression was mostly reduced after miR-302e overexpression (Fig. 4B). Furthermore, the MBNL3 protein expression declined after miR-302e overexpression (Fig. 4C). MiR-302e exhibited lower expression in AGS and MKN45 cells (Fig. 4D). The binding sites among miR-302e and MBNL3 were displayed in Fig. 4E. Moreover, the luciferase activity of MBNL3-WT reporter vectors was reduced, but that of MBNL3-MUT reporter vectors was not changed (Fig. 4F), indicating that miR-302e targeted MBNL3. The cell proliferation was weakened after miR-302e mimic, but this change was offset after MBNL3 overexpression (Fig. 4G). The cell invasion ability was relieved after miR-302e mimic, but this effect was rescued after MBNL3 upregulation (Fig. 4H). Also, the angiogenic ability was attenuated after miR-302e mimic, but this change was reversed after MBNL3 overexpression (Fig. 4I). To sum up, miR-302e targeted MBNL3 to modulate GAC progression.

### MBNL3 Modulated the AKT/VEGFA Pathway to Aggravate GAC Progression

**Fig. 5:**
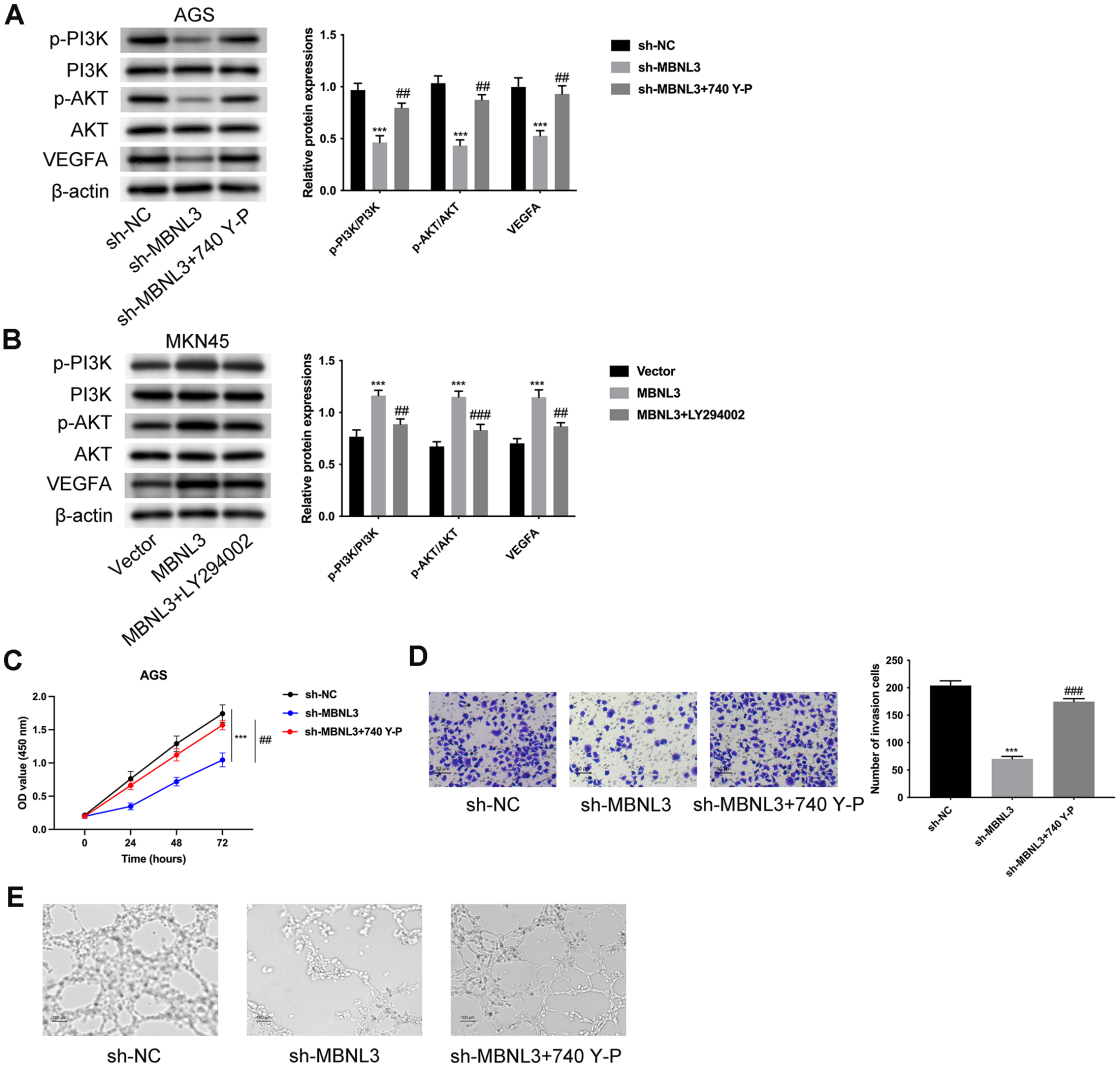
MBNL3 modulated AKT/VEGFA pathway to aggravate GAC progression. (**A**) The protein expressions of p-PI3K, PI3K, p-AKT, AKT, VEGFA were tested through western blot in AGS cells. Groups were divided into the sh-NC, sh- MBNL3 and sh-MBNL3+740 Y-P group. (**B**) The protein expressions of p-PI3K, PI3K, p-AKT, AKT, VEGFA were tested through western blot in MKN45 cells. Groups were divided into the vector, MBNL3, and MBNL3+LY294002 group. (**C**) The cell proliferation was assessed through CCK-8 assay. Groups were divided into the sh-NC, sh-MBNL3 and sh-MBNL3+740 YP group. (**D**) The cell invasion was examined through Transwell assay. Groups were divided into the sh-NC, sh-MBNL3 and sh- MBNL3+740 Y-P group. (**E**) The angiogenic ability was evaluated through tube formation assay. Groups were divided into the sh-NC, sh-MBNL3 and sh-MBNL3+740 Y-P group. ***p < 0.001 vs the sh-NC group; ##*p* < 0.01, ###*p* < 0.001 vs the sh- MBNL3 group.

The levels of p-PI3K/PI3K, p-AKT/AKT, and VEGFA were discovered to be lower after miR-302e overexpression (Fig. S1). The levels of p-PI3K/PI3K, p-AKT/AKT, and VEGFA were all decreased after MBNL3 knockdown, but these effects were reversed by treatment with 740 Y-P (AKT pathway activator) (Fig. 5A). Additionally, the levels of p-PI3K/PI3K, p-AKT/AKT, and VEGFA were increased after MBNL3 overexpression, but these changes were attenuated by treatment with LY294002 (AKT pathway inhibitor) (Fig. 5B). The decreased cell proliferation mediated by MBNL3 suppression was rescued by 740 Y-P treatment (Fig. 5C). Moreover, cell invasion was weakened after silencing MBNL3, but this effect was offset by 740 Y-P treatment (Fig. 5D). The angiogenic ability was reduced after MBNL3 inhibition, but this change was reversed by 740 Y-P treatment (Fig. 5E). In general, MBNL3 modulated the AKT/VEGFA pathway to aggravate GAC progression.

### Suppression of MBNL3 Inhibited Tumor Growth

**Fig. 6:**
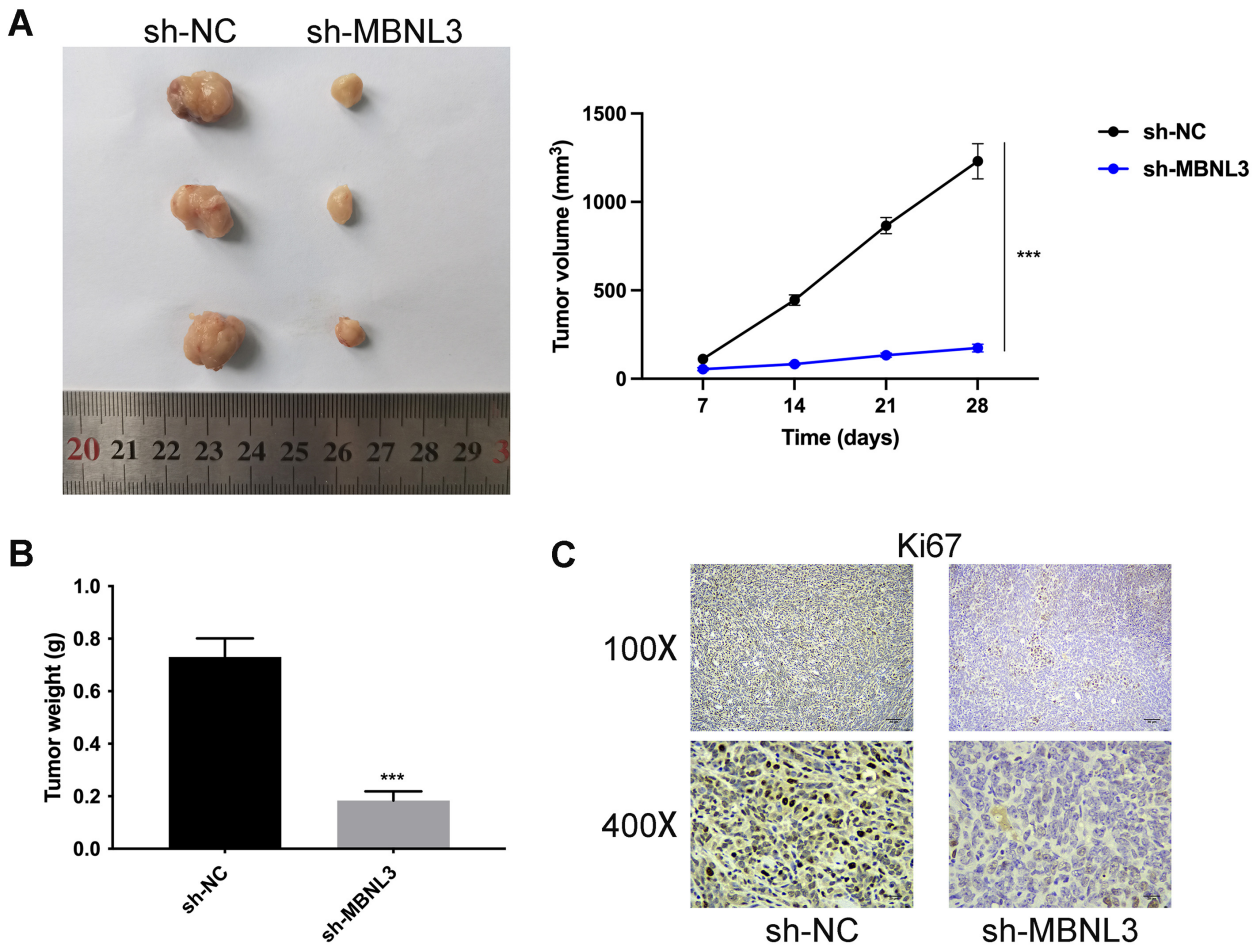
Suppression of MBNL3 inhibited tumor growth. (**A**) The tumor size was assessed after suppressing MBNL3. (**B**) The tumor weight was detected after silencing MBNL3. (**C**) The protein expression of Ki67 was evaluated after inhibiting MBNL3 through IHC assay. ***p < 0.001.

To further probe the regulatory effects of MBNL3 knockdown in GAC cells in vivo, a mouse subcutaneous tumor model was established. In Fig. 6A and 6B, the tumor size, volume, and weight were all decreased after MBNL3 suppression. In addition, through IHC assay, the Ki67 protein expression was reduced after MBNL3 knockdown (Fig. 6C). These data indicated that suppression of MBNL3 inhibited tumor growth in vivo.

## Discussion

Some studies have proved that MBNL3 is involved in regulating the progression of some cancers through serving as a facilitator [6-9]. Nevertheless, the regulatory functions of MBNL3 in GAC remain vague. In this work, we demonstrated that MBNL3 exhibited higher expression, and GAC patients with higher MBNL3 expression had a worse prognosis.

The angiogenesis has been shown to be a critical indicator in GAC progression [14, 15]. Many researchers focus on investigating the angiogenic process in gastric cancer progression. For instance, miR-574-5p targets PTPN3 to enhance angiogenesis in gastric cancer [16]. JP3 (an antiangiogenic peptide) modulates the TRIM25/SP1/MMP2 axis to suppress tumor growth, metastasis, and angiogenesis in gastric cancer [17]. Additionally, suppression of PAX3 affects the MET/PI3K axis to inhibit cell proliferation and angiogenesis in gastric cancer [18]. In addition, silencing of MED27 retards the Wnt/β-catenin pathway to retard metastasis and angiogenesis in gastric cancer [19]. Similar to these studies, this work demonstrated that overexpression of MBNL3 facilitated, while knockdown of MBNL3 suppressed cell proliferation, invasion, and angiogenesis in GAC.

The miR-mRNA axis has been verified to be a hackneyed regulatory mechanism in the progression of cancers, including gastric cancer. For instance, miR-877 downregulates AQP3 to retard gastric cancer progression [20]. Moreover, miR-613 modulates PFKFB2 to relieve the Warburg effect in gastric cancer [21]. MiR-489 targets HDAC7 to suppress the development of gastric cancer through the PI3K/AKT pathway [22]. It has been discovered that miR-302e exhibits a suppressive role in various cancers. For example, miR-302e affects VEGFA expression to inhibit glioma progression [23]. The lncRNA FGD5-AS1/miR-302e/CDCA7 axis aggravates cell proliferation, migration, and invasion in colorectal cancer [24]. Furthermore, miR-302e targets CXCL1 to modulate cell proliferation, invasion, and apoptosis of colorectal cancer [25]. However, the functions of miR-302e on the progression of GAC remain vague. In this study, we illustrated that miR-302e combined with and targeted MBNL3, thereby forming the miR-302e/MBNL3 axis in GAC. Next, rescue assays revealed that miR-302e targeted MBNL3 to modulate GAC progression.

The AKT pathway has been shown to participate into the regulation of gastric cancer. For example, ORAI2 modulates PI3K/AKT signaling to aggravate tumor growth and metastasis in gastric cancer [26]. miR-21 affects the PI3K/Akt/mTOR pathway to regulate cisplatin resistance and autophagy in gastric cancer [27]. Moreover, NUP37 facilitates the PI3K/AKT/mTOR pathway to accelerate tumorigenesis in gastric cancer [28]. Similar to these studies, MBNL3 was also found in our study to activate the AKT/VEGFA pathway. In addition, the regulatory effects of MBNL3 knockdown on GAC cell proliferation, invasion, and angiogenesis were reversed after 740 Y-P (AKT activator) treatment. Through in vivo assay, it was proved that inhibition of MBNL3 slowed tumor growth in vivo.

In conclusion, for the first time, this work highlighted the regulatory functions of MBNL3 in GAC progression. MBNL3 was revealed to act as a target of miR-302e to facilitate cell proliferation, invasion, and angiogenesis of gastric adenocarcinoma through the AKT/VEGFA pathway. Nevertheless, some limitations also exist, such as lacking experiments on other phenotypes (autophagy, exosome, immune response). In the future, more experiments will be done to study the regulatory roles of MBNL3 in the progression of GAC.

## Supplemental Materials

Supplementary data for this paper are available on-line only at http://jmb.or.kr.
