# Supplementary figures and images for: MBNL3 Acts as a Target of miR-302e to Facilitate Cell Proliferation, Invasion and Angiogenesis of Gastric Adenocarcinoma via AKT/VEGFA Pathway
Source: J Microbiol Biotechnol. 2024 May 30;34(7):1433–42. doi: 10.4014/jmb.2401.01027 (PMC11294653; doi:10.4014/jmb.2401.01027)

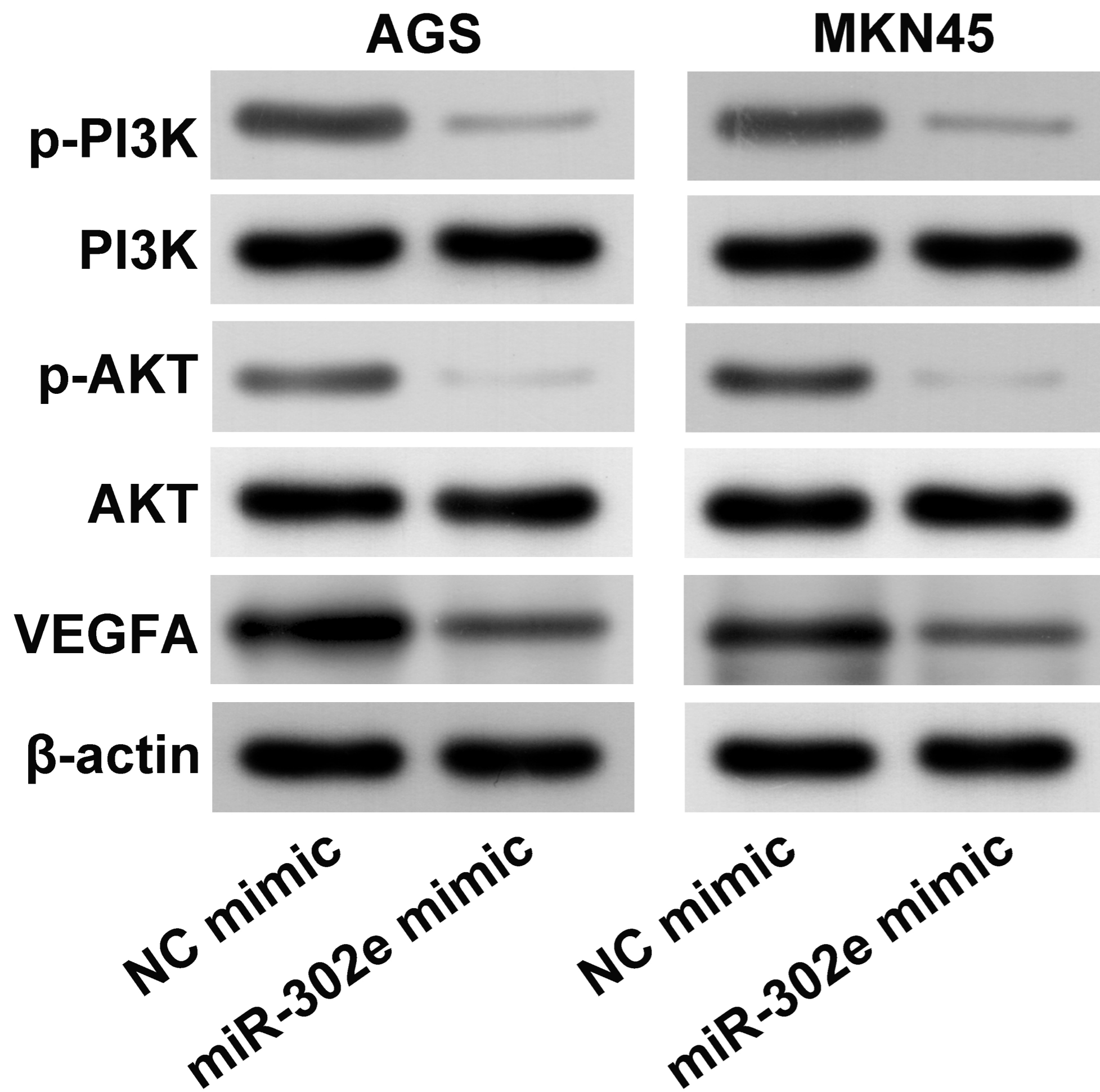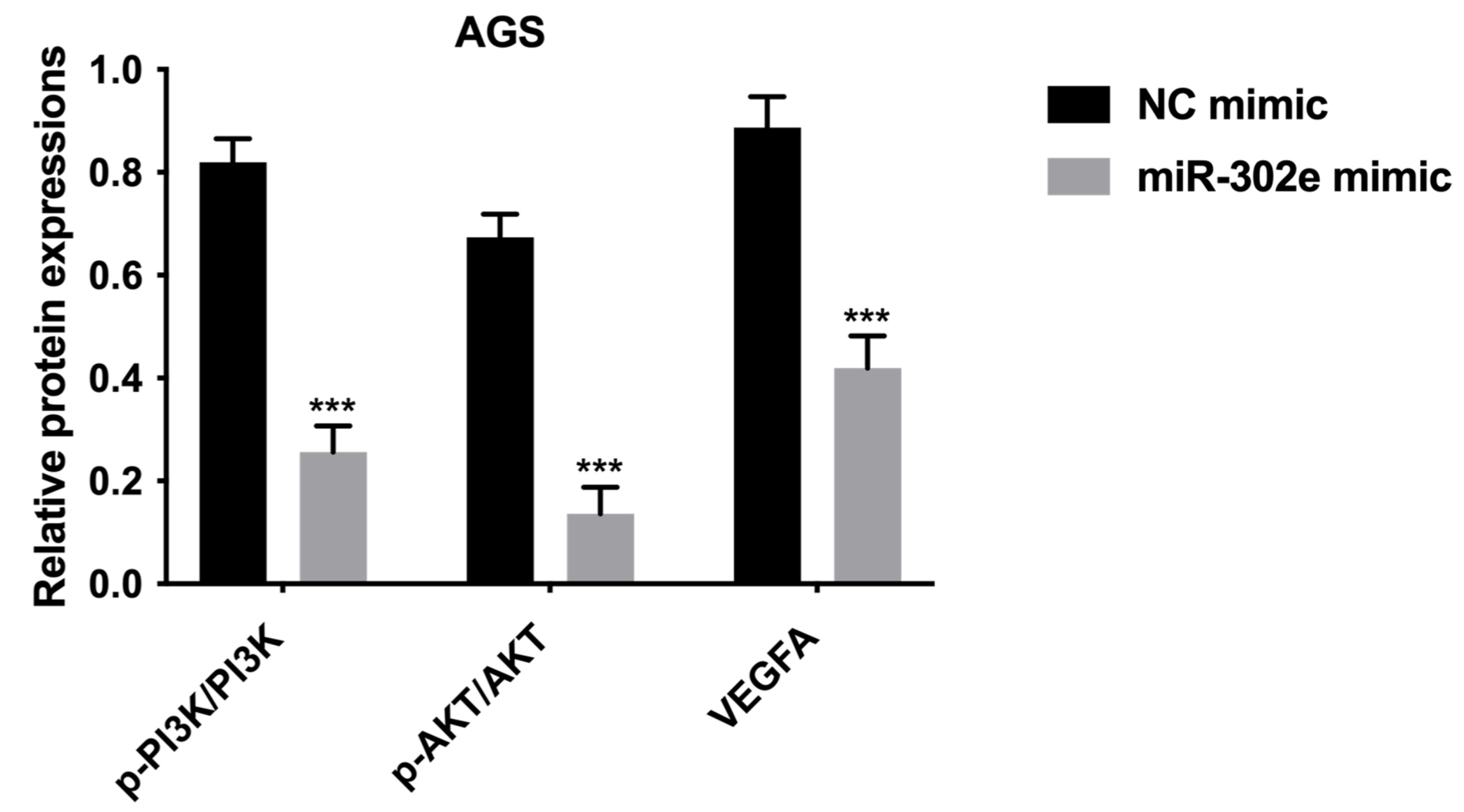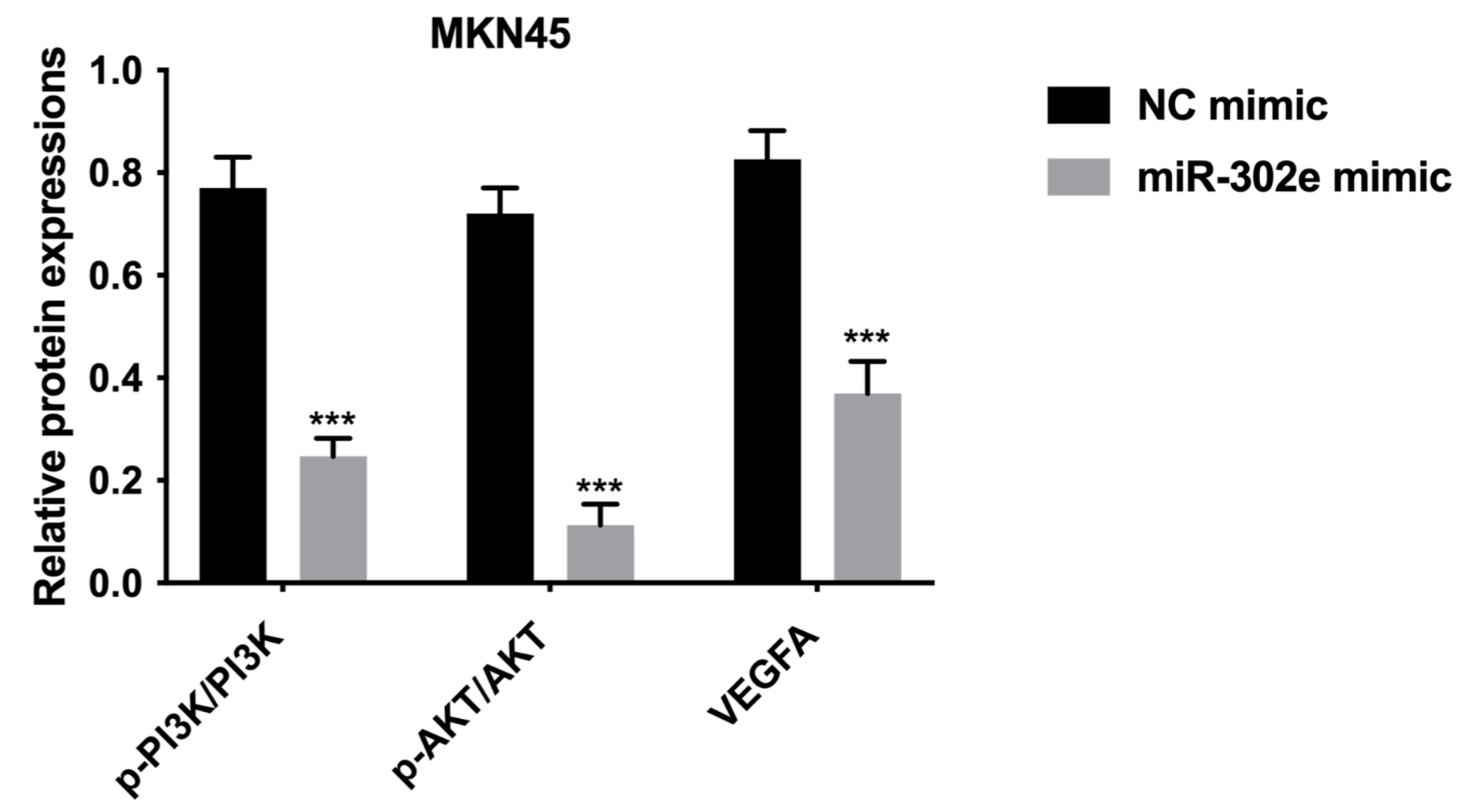

Supplement: Supplementary file 1 [file jmb-34-7-1433-supple.pdf]
